# Endoscopic Intrapyloric Botulinum Toxin Injection with Pyloric Balloon Dilation for Symptoms of Delayed Gastric Emptying after Distal Esophagectomy for Esophageal Cancer: A 10-Year Experience

**DOI:** 10.3390/cancers14235743

**Published:** 2022-11-23

**Authors:** Manoop S. Bhutani, Shamim Ejaz, Irina M. Cazacu, Ben S. Singh, Mehnaz Shafi, John R. Stroehlein, Reza J. Mehran, Garrett Walsh, Ara Vaporciyan, Stephen G. Swisher, Wayne Hofstetter

**Affiliations:** 1 Department of Gastroenterology, Hepatology and Nutrition, The University of Texas MD Anderson Cancer Center, Houston, TX 77030, USA; 2 Department of Thoracic and Cardiovascular Surgery, The University of Texas MD Anderson Cancer Center, Houston, TX 77030, USA

**Keywords:** esophagectomy, esophageal cancer, gastroparesis, delayed gastric emptying, botulinum toxin, botox, pyloric dilation

## Abstract

**Simple Summary:**

Delayed gastric emptying is a disorder in which the stomach doesn’t empty food and fluids as fast as it normally should. This condition is common following the removal of the esophagus for treatment of esophageal cancer. One option for the treatment of this motility disorder include the injection of botulinum toxin into the muscular ring at the opening of the stomach into the first part of the small intestine or inflating a long balloon in the opening between the stomach and small intestine to improve the emptying of the stomach. Our aim was to investigate whether combining these two treatment methods would enhance the overall therapeutic benefits to treat delayed gastric emptying.

**Abstract:**

Patients with esophageal cancer undergoing esophagectomy have an improved survival over time, however adverse events associated with the use of a gastric conduit are increasingly being reported. Delayed gastric emptying (DGE) is an esophagectomy-related complication which can decreased quality of life by causing debilitating gastrointestinal symptoms and malnutrition. The aim of our study was to evaluate the effect of endoscopic intrapyloric botulinum (BT) injection in combination with pyloric balloon dilation in patients with DGE following distal esophagectomy at our tertiary cancer center. Patients with a prior history of distal esophagectomy who had also undergone endoscopic BT injection with pyloric balloon dilation by a single endoscopist between 2007 and 2017 were included in the study. One hundred units of BT were injected endoscopically into the pylorus in four quadrants using an injection needle. Following BT injection, a standard through-the-scope balloon was passed to the pylorus and inflated to a maximum diameter of 12–20 mm. For patients who underwent repeat procedures, the symptomatic outcomes were assessed and documented by the endoscopist; for the other patients, the electronic medical records were reviewed. A total of 21 patients undergoing 44 endoscopic intrapyloric botox injections combined with balloon dilatations were identified. The patients underwent the procedures at a median of 22 months (range, 1–108 months) after esophagectomy. The procedures were performed only once in 43% of the patients; 43% patients underwent the procedure twice, while 14% had it multiple times (>2). Overall, intrapyloric BT injection coupled with balloon dilation was a safe procedure, without any major immediate or delayed (1 month) procedure-related adverse events. Eighteen patients (85%) reported a significant overall improvement in symptoms from the initial presentation. One patient (5%) showed no improvement, whereas in two (10%) patients responses were not available. In our particular cohort of patients, the interventions of endoscopic intrapyloric BT injection with pyloric balloon dilation proved to be very beneficial, leading to significant symptomatic improvement. The balloon dilation after BT injection might have resulted in better diffusion of the BT into the pyloric sphincter complex, possibly increasing its therapeutic effects. Further prospective studies are needed to validate these results.

## 1. Introduction

Surgical resection for distal esophageal cancer is performed with curative intent or for palliation of dysphagia. The stomach is most commonly used to restore the continuity of the gastrointestinal tract after distal esophagectomy by mobilizing a gastric conduit to the thorax and creating an anastomosis to the remaining part of the esophagus [1]. As patients with esophageal cancer have an improved survival over time, complications associated with the use of a gastric conduit are increasingly being reported [2]. One of the adverse events related to esophagectomy is delayed gastric emptying, which may cause debilitating symptoms and put the patients at risk for malnutrition and decreased quality of life [3]. 

To prevent delayed gastric emptying after distal esophagectomy, pyloric drainage procedures such as pyloroplasty or pyloromyotomy have been performed. However, their benefit in the context of esophagectomy still remains a matter of debate, and randomized trials have not been able to consistently document their clinical usefulness [1,4,5]. Moreover, these procedure are associated with duodenal leak and possibly an increase in dumping syndrome and bile reflux [6,7]. The intrapyloric injection of botulinum toxin (BT or botox) during esophageal surgery has been used as an alternative to surgical drainage procedures. The BT injection has an inhibitory effect on the smooth muscle of the gastrointestinal tract and relaxes the pylorus during the early postoperative period [8]. However, regarding long-term results, a study has shown that patients who received intra-pyloric BT injection during surgery had significantly more reflux symptoms and required frequent post-operative endoscopic pylorus dilatations [9]. Several studies have evaluated the effect of balloon dilation of the pylorus after esophagectomy to improve gastric emptying, with balloon diameters often ranging from 12–22 mm [10,11,12]. The aim of our study was to evaluate the effect of endoscopic intrapyloric BT injection with subsequent pyloric balloon dilation on post-esophagectomy delayed gastric emptying in patients with esophageal carcinoma who were treated by a single endoscopist at our tertiary cancer center.

## 2. Methods

After approval from the institutional review board, we retrospectively reviewed the medical records of the patients with a prior history of esophageal cancer and esophagectomy who had undergone endoscopic BT injection with pyloric balloon dilation to relieve the symptoms of delayed gastric emptying at our institution by a single endoscopist (MSB) between 2007 and 2017. Data were retrospectively extracted from our endoscopy records (prospectively maintained electronic endoscopic database) and patients' hospital electronic medical records and included demographics, esophageal cancer histological type and staging, type of surgery, symptoms of delayed gastric emptying, results of imaging studies, technical details of endoscopic BT injection and balloon dilation, procedure-related adverse events, and outcomes of the procedures (for patients who underwent repeat procedures, the outcomes were assessed and documented by the endoscopist; for the other patients, the electronic medical records were reviewed). A descriptive statistical analysis was performed. The distribution of continuous variables was summarized by means and standard deviations. The distribution of categorical variables was summarized using frequencies and percentages. All statistical evaluations were two-sided, and a P-value of <0.05 was considered statistically significant. A statistical analysis was carried out using SPSS Statistics software (version 24.0; IBM Corporation, Armonk, NY, USA).

### 2.1. Endoscopic Injection of Botulinum Toxin

All procedures were performed under general anesthesia with endotracheal intubation after overnight fasting. After complete diagnostic examination including ruling out recurrent esophageal cancer or other mechanical etiology, a clear view of the pylorus was obtained from the antrum. A botulinum toxin vial of 100 units(U) was mixed with 4 cc of normal saline and reconstituted. Using a standard endoscopic injection needle (25G), twenty five units of botulinum/mL was then injected into each of four quadrants of the pylorus.

### 2.2. Pyloric Balloon Dilation 

Following BT injection, a standard through-the-scope pyloric balloon was passed through the pylorus under direct endoscopic view and inflated to a maximum diameter of 12–20 mm until significant resistance was encountered. The starting size of the balloon was determined by the endoscopist based on an assessment of the pyloric tone and inflated to a size where mild to moderate resistance was appreciated. At that point the balloon inflation was held for one minute. 

**Figure 1:**
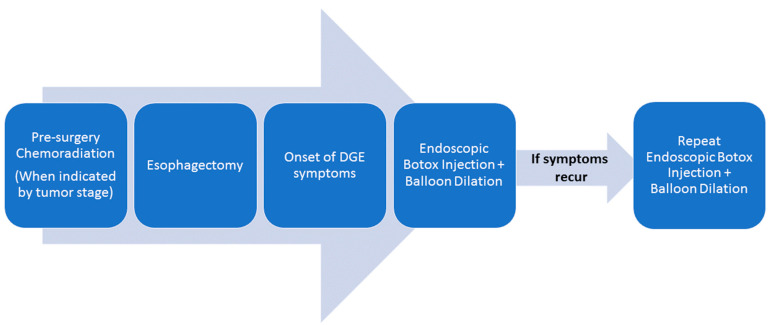
Treatment course for delayed gastric emptying.

All endoscopic procedures were performed by the same endoscopist (MSB) using a similar technique. In patients who had a repeat BT injection, the same endoscopist queried the patient about the estimated percentage improvement in symptoms prior to each repeat procedure and documented this data in the patients’ chart. Repeat BT endoscopic injections were performed on demand at the time frame where the patients who had a significant improvement in their symptoms felt that the response had waned and that it was time for another injection. The full treatment course that every patient experienced can be visualized in Figure 1. 

## 3. Results

A total of 21 patients who had undergone endoscopic BT injection and pyloric balloon dilation for delayed gastric emptying after esophagectomy were identified (Table 1). The median age was 58 years (range, 30–73) and most patients were males (81%). Regarding cancer history, 81% had esophageal cancer and 19% had cancer of the gastro-esophageal junction. A majority of patients (91%) were diagnosed with esophageal adenocarcinoma, while 9% of patients had squamous cell carcinoma. 

Regarding surgical resection, 52% of patients had Ivor-Lewis esophagectomy, 38% had Ivor-Lewis esophagectomy with pyloroplasty, and 10% had Ivor-Lewis esophagectomy with pyloromyotomy. Of the 21 surgical procedures, 14 (66.7%) were performed open and 7 (33.3%) were minimally invasive (Table 2). 

All patients had DGE symptoms at the time of intervention. Early satiety was present in all patients. In our study cohort, 10 of 21 patients (48%) were seen to have received either metoclopramide or erythromycin as a pharmacotherapy treatment for their DGE. In addition, vomiting (67%), aspiration (24%), chest pain (24%), regurgitation (57%) and dysphagia (52%) were other common presentations. Additional objective evidence consistent with delayed gastric emptying was obtained by radiographic studies such as esophagram showing retention of contrast in the gastric conduit (14%) or by the presence of food or retained fluid in the stomach at the time of endoscopy (86%) despite an overnight fast.

In total, 44 endoscopic intrapyloric BT injections were performed. The patients underwent the procedure at a median of 22 months (range, 1–108 months) after esophagectomy. Pyloric balloon dilations were performed at the time of botox procedures for all patients, using controlled radial expansion balloon dilators ranging from 12 to 20 mm. 

BT injection with balloon dilation was performed only once in 43% of patients; 43% underwent the procedures twice, while 14% of patients had it multiple times (>2). Interestingly, 70% (7/10) of the patients who had undergone a pyloroplasty or pyloromyotomy during esophagectomy only required a single endoscopic BT injection with balloon dilation. The remaining 30% (3/10) needed only a single repeat procedure with an average 8 month period in between procedures. In contrast, more patients (8/11, 73%) were observed to require multiple endoscopic BT injection with balloon dilation for those who did not have a pylorus altering procedure during Ivor-Lewis esophagectomy. The remaining patients (3/11, 27%) only needed a single treatment procedure to alleviate the delayed gastric emptying symptoms.

Overall, intrapyloric BT injection coupled with balloon dilation was a safe procedure, without any major immediate or delayed (1 month) procedure-related adverse events. Only one patient complained of shortness of breath after the procedure and was found to be hypoxic. The patient had chronic obstructive pulmonary disease exacerbation triggered by aspiration pneumonitis. He had retained food in the stomach at the time of endoscopy and developed aspiration pneumonitis despite upfront endotracheal intubation. The patient recovered and was discharged after two days of hospitalization.

The outcome of the intervention of BT injection with balloon dilation for patients who only had a single injection with dilation was based on the review of electronic medical records regarding the post-procedure follow-up visits with the referring physician. For patients undergoing repeat procedures, the outcomes were documented by the endoscopist based on patient interview prior to performing the repeat injection. Eighteen patients (85%) reported improvement in symptoms for more than 50% from the initial presentation. One patient (5%) showed no improvement, whereas in two (10%) patients responses were not available. 

## 4. Discussion

Delayed gastric emptying is one of the major complications occurring in patients after esophagectomy. According to a systematic review, the overall reported incidence of delayed gastric emptying after esophagectomy was in the range of 2.2–47% [13]. The definition of postoperative delayed gastric emptying varied across the 25 studies included in the review, and it was based on clinical, radiological or combined criteria. However, symptoms of delayed gastric emptying have been reported to occur in over 50% of patients after esophagectomy [2]. Delayed gastric emptying can cause symptoms of nausea, vomiting and early satiety. Furthermore, patients may develop chronic reflux and aspiration pneumonia, which may have a significant effect on the quality of life.

Bilateral vagotomy, increased pyloric resistance and reduced stomach capacity are the major delayed gastric emptying pathogenesis mechanisms [2,14]. Structures that limit reflux are usually removed during surgery. In addition, division of the vagus nerves leads to dysmotility of the stomach and delayed emptying due to pylorospasm [15]. Various pyloric drainage procedures can be performed during esophagectomy intraoperatively such as pyloromyotomy, pyloroplasty, pyloric balloon dilation, pyloric injection of botulinum toxin or a combination of balloon dilation with botulinum toxin injection to decrease the incidence of delayed gastric emptying and other symptoms [16]. The focus of this report, however, is the use of intrapyloric botulinum toxin injection in combination with balloon dilation in the post-operative period at various time frames when there was symptomatic clinical and other objective evidence of delayed gastric emptying. 

Botulinum toxin is a bacterial neurotoxin that releases acetylcholine into the neuromuscular junction, thus inhibiting muscle contraction. An intrapyloric botulinum toxin injection is able to reduce pyloric motor activity and to accelerate gastric emptying in patients with delayed gastric emptying [17] . The intrapyloric injection of BT has been one of the most widespread endoscopic pyloric-directed therapies for delayed gastric emptying [18]. It was initially used in the gastrointestinal tract as a treatment for achalasia [19]. Several open label clinical trials of pyloric injections of BT in non-esophagectomized patients with gastroparesis showed good results with significant symptomatic improvement [20,21,22,23]. However, two randomized clinical trials in patients with native stomachs without esophagectomy showed some improvement in gastric emptying and symptoms after intrapyloric injections of BT but these responses were not significantly different when compared to placebo [24,25]. The results of these studies formed the basis of a recommendation of the American College of Gastroenterology guidelines in 2013 and recently in 2022 against the use of BT injections for gastroparesis [16,26]. Pasricha et al. have also recently discouraged its use outside of research trials [27]. However, despite these recommendations, the use of intrapyloric botulinum toxin injection in clinical practice remains widespread due to limited alternatives and good safety profile. Open label non-randomized studies have shown that BT injections may still benefit a subset of patients with gastroparesis [28,29] despite the negative RCTs. 

In the post-esophagectomy patients, the study by Nevins et al. showed 100% resolution of symptoms with intrapyloric botulinum toxin injection and 20% needing repeat injection [30]. Similarly, Reichenbach et al. reported 64% of their patient cohort had achieved symptomatic improvement at 1 month following BT injection into the pylorus, with improvements lasting up to 6 months [31]. Furthermore, recent studies evaluating the therapeutic effects of pyloric balloon dilation for DGE showed a response rate of 53% at 2 months following dilation [32]. Another recent early post-operative DGE study investigating pyloric dilations used 30mm balloons to achieve a success rate of 58% [33]. The reason we decided to start combining pyloric dilation with botulinim toxin injection was that although we did not do a formal study, it was our impression at our institution that BT alone was not providing the remarkable results as seen by Nevins et al of 100 percent response. Thus we decided to combine two therapeutic procedures together in order to improve the response rate. However, the true additional benefit of balloon dilation in addition to botulinum toxin injection can only be proven in a randomized trial with botox alone versus botox with balloon dilation in the same group of patients. 

In the current study, we shared our retrospective review of 10 year duration of pyloric BT injection in combination with pyloric balloon dilation, with an evidence of significant symptomatic benefit in 85% of patients. Repeat injection with balloon dilation was done only on demand and at the request of the patient in 67% of patients. All of these patients were interviewed by the endoscopist prior to injection and the patients clearly felt that there was significant improvement in their symptoms with the prior injection and by the time they requested a repeat injection there was a relapse of their symptoms to the pre-injection level. The patients were asked to estimate the amount of symptomatic improvement in their symptoms in percentage from the first injection, which was documented in the patient’s medical record at the time of the repeat injection with balloon dilation. Furthermore, all the initial BT injections with pyloric dilation were performed by the endoscopist (MSB) at the request/referral from a thoracic surgeon or a gastroenterology colleague for significant post esophagectomy symptoms/imaging consistent with delayed gastric emptying, decreasing the chance of a selection bias or preference to do this procedure by the endoscopist. 

Despite the negative randomized clinical trials (RCTs) cited in the ACG guidelines [16,26], there is widespread belief among clinicians that there are subsets of patients with delayed gastric emptying where botulinum toxin injection has a clear benefit that needs more studies and research. The efficacy of intrapyloric BT injections is likely different in our patients due to a number of factors. The above RCTs were done in patients with an intact stomach with diabetic or idiopathic gastroparesis, and not in patients with delayed gastric emptying after esophagectomy. During esophagectomy, many patients in our study underwent pyloroplasty and therefore the basal pyloric tone was likely already lower than a virgin pylorus, and thus the combination of BT injection and pyloric dilation was likely more efficacious as a smaller decrease in pyloric resistance due to our intervention could have resulted in a meaningful clinical benefit. In addition, compared to a native stomach in distal esophagectomy, the stomach is vertically pulled up to the thorax to create a conduit, and therefore gravity plays an additional role in gastric emptying that may potentiate whatever decrease in pyloric resistance that may have been accomplished by the injection of BT and balloon dilation. 

In addition, the RCTs mentioned above did not combine intrapyloric BT injection with pyloric balloon dilation. Our study differs from these RCTs as patients received both endoscopic intrapyloric BT injection and balloon dilatation of the pylorus for post-esophagectomy delayed gastric emptying. A study by Gourceral et al. showed that 10 patients with refractory gastroparesis who received only pyloric dilation had symptomatic benefit and increased quality of life [34]. As mentioned earlier, a recent study of pyloric balloon dilation for DGE showed a response rate of 53% at 2 months [32]. In fact, a recent study, albeit in children, showed a beneficial effect when combining pyloric dilation with pyloric BT injections in patients who have dyspepsia with or without DGE [35]. It is possible that the combination of BT injection with pyloric dilation provides additional therapeutic benefits due to a synergistic effect, and RCTs to study the combined effect of this intervention would be worthwhile given the promising results in this retrospective study of our patients as determined from a prospectively maintained endoscopic database. We hypothesize that the balloon dilation after injection may also result in better diffusion of the botulinum toxin into the pyloric sphincter complex by pushing any botulinum toxin in the submucosal tissue into the muscularis propria and possibly increasing its therapeutic effects. 

There is no definitive explanation for why some patients required multiple endoscopic BT injections, while others only needed a single procedure to improve their delayed gastric emptying symptoms. As mentioned in the results sections, a majority of the patients who had undergone a pyloroplasty or pyloromyotomy required only one procedure. In contrast, the patients who had an unaltered pylorus were observed to need multiple procedures. Having a pyloroplasty or pyloromyotomy may increase the efficacy of endoscopic BT injection and balloon dilation, but it is also possible that patients may have received subsequent endoscopic treatment of delayed gastric emptying at their home or local institution, instead of our tertiary center. Another interesting aspect of our study is the need for BT injection with pyloric dilation occurring at different times from an esophagectomy. The patients underwent the procedures at a median of 22 months (range, 1–108 months) after esophagectomy. We hypothesize that many patients after esophagectomy have already had some change in the gastric emptying, but their symptoms are manageable, and the patients adjust to a new norm after esophagectomy. At some point there is an additional insult to the gastric neuroenteric system. To speculate, this may be due to starting a new medication, a viral gastroenteritis, or another non-viral gastrointestinal illness resulting in a post-infectious hit to the already borderline gastric neuroenteric system. 

Some limitations of our study should be acknowledged, such as its retrospective design or response to treatment assessed by chart review, and not using a validated symptom assessment score. However, the strengths are that a single endoscopist performed all of the procedures, therefore there was consistency in the technique. Other endoscopists at our institution have performed endoscopic BT injection during this time period but we excluded those to decrease technical variables such using a different technique, using a different dose of BT and not combining pyloric dilation with BT so that at least the results could not be questioned based on operator dependency. The combination of intrapyloric balloon dilation with BT injection for treatment of delayed gastric emptying used in this study has not been the focus of prior RCTs. Moreover, at least for the patients who received repeat injections, the same endoscopist questioned them regarding their symptomatic response to the procedure and the duration of improvement as part of a prospectively maintained endoscopic database which was retrospectively reviewed 10 years later. Our group has started a prospective study to systematically assess the symptomatic treatment response after BT injections coupled with balloon dilation, using validated scales of Gastroparesis Cardinal Symptom Index (GCSI) and quality of life. 

## 5. Conclusions

In our particular cohort of patients, the combination of intrapyloric balloon dilation with BT injection for the treatment of delayed gastric emptying after esophagectomy proved to be a safe procedure, with evidence of significant symptomatic benefit in 85% of patients. The balloon dilation after BT injection potentially resulted in better diffusion of the BT into the pyloric sphincter complex and possibly increased its therapeutic effects. Our ongoing prospective study assessing the symptomatic treatment response after BT injections coupled with balloon dilation is expected to offer better insights into the effect of these procedures on post-esophagectomy delayed gastric emptying symptoms in patients with esophageal carcinoma. A prospective randomized controlled trial of a combination of BT with pyloric dilation in post esophagectomy patients would be ideal. 

## Figures and Tables

**Table 1:** Baseline Characteristics of Study Population.

Gender, No. Patients	Percent
Male	81%
Female	19%
**Age, Mean year (range)**	58 (30–73)
**Cancer History**	
Esophageal Cancer	81%
GE Junction Cancer	19%
Esophageal Adenocarcinoma	91%
Esophageal Squamous Cell Carcinoma	9%
**Symptoms**	
Early Satiety	100%
Vomiting	67%
Aspiration	24%
Chest Pain	24%
Regurgitation	57%
Dysphagia	52%
**Evidence of delayed gastric emptying**	
Esophagram	3 (14%)
Endoscopic evidence of retained food/large amount of fluid in the stomach	18 (86%)

**Table 2:** Surgical characteristics of study population.

**Surgical Resection (*n* = 21)**	***n* (Percent)**
Ivor-Lewis esophagectomy	11 (52%)
Ivor-Lewis esophagectomy with pyloroplasty	8 (38%)
Ivor-Lewis esophagectomy with pyloromyotomy	2 (10%)
Open surgery	14 (67%)
Minimally invasive	7 (33%)
**Tumor Staging**	
T1a/1b	4 (19%)
T2	2 (10%)
T3/T4 *	15 (71%)

* Only 1 patient was staged T4.

## Data Availability

The data presented in this study are available on request from the corresponding author.
